# A Parabrachial-Hypothalamic Cholecystokinin Neurocircuit Controls Counterregulatory Responses to Hypoglycemia

**DOI:** 10.1016/j.cmet.2014.11.006

**Published:** 2014-12-02

**Authors:** Alastair S. Garfield, Bhavik P. Shah, Joseph C. Madara, Luke K. Burke, Christa M. Patterson, Jonathan Flak, Rachael L. Neve, Mark L. Evans, Bradford B. Lowell, Martin G. Myers, Lora K. Heisler

**Affiliations:** 1 Centre for Integrative Physiology, Hugh Robson Building, University of Edinburgh, Edinburgh, EH8 9XD, UK; 2 Division of Endocrinology, Diabetes and Metabolism, Department of Medicine, Beth Israel Deaconess Medical Center, Harvard Medical School, Boston, MA 02115, USA; 3 Department of Medicine and Wellcome Trust/Medical Research Council Institute of Metabolic Science, University of Cambridge, Cambridge, CB2 0QQ, UK; 4 Rowett Institute of Nutrition and Health, University of Aberdeen, Aberdeen, AB25 2ZD, UK; 5 Division of Metabolism, Endocrinology, and Diabetes, Department of Internal Medicine, University of Michigan, Ann Arbor, MI 48105, USA; 6 Picower Institute for Learning and Memory, Department of Brain and Cognitive Sciences, Massachusetts Institute of Technology, Cambridge, MA 02139, USA

## Abstract

Hypoglycemia engenders an autonomically mediated counterregulatory (CR)-response that stimulates endogenous glucose production to maintain concentrations within an appropriate physiological range. Although the involvement of the brain in preserving normoglycemia has been established, the neurocircuitry underlying centrally mediated CR-responses remains unclear. Here we demonstrate that lateral parabrachial nucleus cholecystokinin (CCK^LPBN^) neurons are a population of glucose-sensing cells (glucose inhibited) with counterregulatory capacity. Furthermore, we reveal that steroidogenic-factor 1 (SF1)-expressing neurons of the ventromedial nucleus of the hypothalamus (SF1^VMH^) are the specific target of CCK^LPBN^ glucoregulatory neurons. This discrete CCK^LPBN^→SF1^VMH^ neurocircuit is both necessary and sufficient for the induction of CR-responses. Together, these data identify CCK^LPBN^ neurons, and specifically CCK neuropeptide, as glucoregulatory and provide significant insight into the homeostatic mechanisms controlling CR-responses to hypoglycemia.

## Highlights

• CCK^LPBN^ neurons are glucose inhibited and activated by hypoglycemia
• CCK^LPBN^ neurons are necessary and sufficient for counterregulatory (CR)-responses
• CCK neuropeptide is the key mediator of CCK^LPBN^ neuron-mediated CR-responses
• CCK^LPBN^ neuron-induced CR-responses require downstream SF1^VMH^ neurons

## Graphical Abstract

**Figure:**
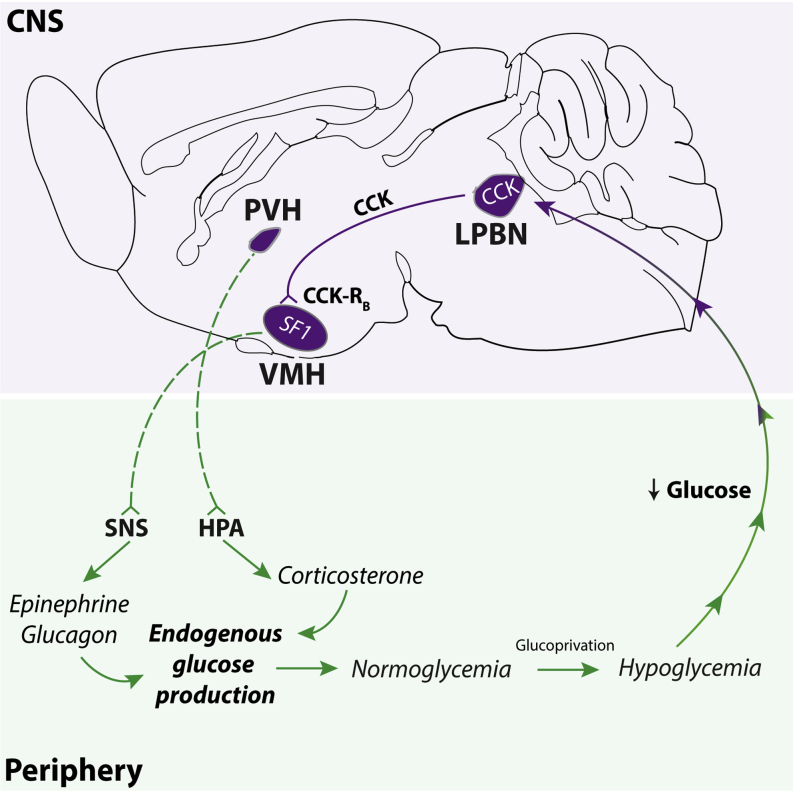

## Teaser

The counterregulatory response (CRR) to hypoglycemia is critical for the maintenance of normoglycemia and governed by the brain. Garfield et al. identify a population of brainstem CCK neurons that directly sense extracellular glucose concentrations and, via their connection to SF1 hypothalamic neurons, promote CRR.

## Introduction

Due to the serious pathophysiological consequences of low blood glucose, normoglycemia is a tightly defended state regulated by a conserved and coordinated network of peripheral and central systems. Sensory information from peripheral glucosensors is integrated into the wider glucoregulatory/sensory circuitry, defined principally by the brainstem and hypothalamus, which in turn engages the sympathetic nervous system (SNS) and hypothalamic-pituitary-adrenal axis to stimulate glucose production and inhibit glucose uptake (Marty et al., 2007). Yet, it is unclear how these disparate structures converge to orchestrate sensorimotor responses to dysglycemia.

The lateral parabrachial nucleus (LPBN) forms part of the preautonomic circuitry that subserves physiological responses to numerous viscerosensory modalities, including nociception (Hermanson et al., 1998), thermostasis (Nakamura and Morrison, 2008), malaise (Carter et al., 2013), and energy homeostasis (Wu et al., 2012). As an assimilatory interoceptive relay for ascending sensory information, the LPBN is therefore neuroanatomically positioned to respond to various aspects of homeostatic dysregulation (Saper, 2002). Indeed, the LPBN has been implicated in responses to hypoglycemia (Briski, 1999, Fujiwara et al., 1988, Ritter and Dinh, 1994), but detailed characterization has hitherto been lacking.

## Results and Discussion

### CCK^LPBN^ Neurons Are Responsive to CR-Stimuli

**Figure 1:**
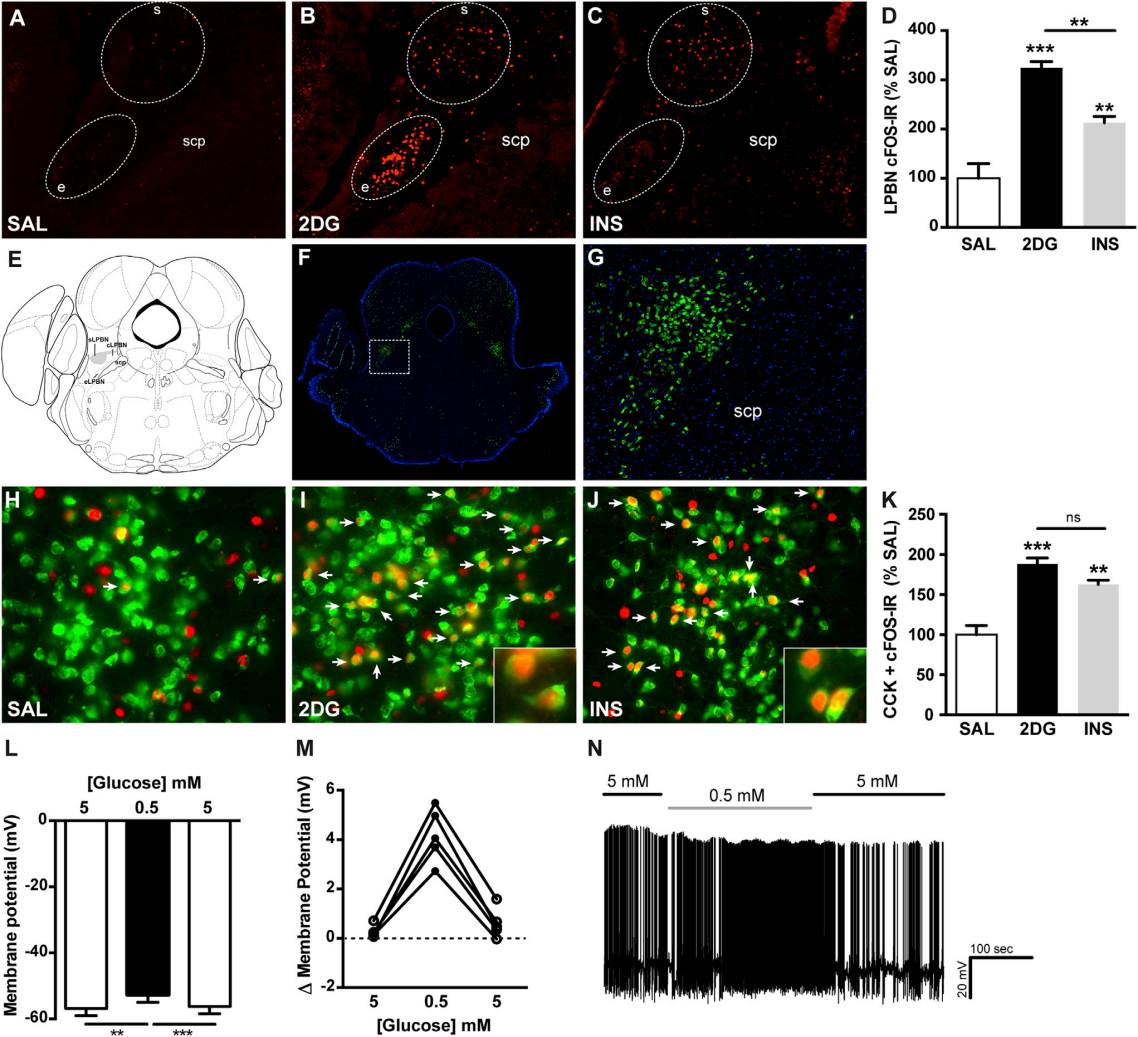
CCK^LPBN^ Neurons Are Activated by Glucoprivation (A–D) 2DG- and INS-induced glucoprivation promoted cFOS-IR (red) within the LPBN, as compared to saline controls. Both glucoprivic stimuli elicited cFOS-IR within the sLPBN (B,C), with 2DG also increasing neural activity within the external compartment of the LPBN (B). (D) Quantification of cFOS-IR across the rostral-to-caudal extent of the LPBN (see Figure S1) revealed a significant elevation of cFOS-IR in 2DG- and INS-treated mice above saline controls (n = 3–5 per group; one-way ANOVA, F_(2,8)_ = 31.1, p = 0.0002 with Tukey’s post hoc comparison). (E–G) The sLPBN is located at the rostral and dorsal extreme of the LPBN and defined by the expression of CCK. (F and G) Transgenic labeling of CCK neurons (green) in a *CCK-ires-Cre::R26-loxSTOPlox-L10-GFP* mouse line recapitulated the known endogenous expression profile. (H–K) 2DG- and INS-induced glucoprivation increased cFOS-IR (red) within CCK^LPBN^ neurons (green) compared to saline controls (white arrows denote colocalized neurons) (n = 3–5 per group; one-way ANOVA, F_(2,9)_ = 23.2, p = 0.0003 with Tukey’s post hoc comparison). (L–N) A subset of CCK^LPBN^ neurons were inhibited by glucose. (L and M) 5/14 CCK^LPBN^ neurons exhibited reversible membrane depolarization in response to a downward glucose step form 5 mM to 0.5 mM (n = 5, repeated-measures one-way ANOVA, F_(2,4)_ = 77.3, p = 0.0003 with Tukey’s post hoc comparison). (N) Representative electrophysiological trace from a spontaneously active glucose-inhibited CCK^LPBN^ neuron. 2DG, 2-deoxyglucose; e, external LPBN; INS, insulin; SAL, saline; scp, superior cerebellar peduncle; s, superior LPBN. All data are presented as mean ± SEM. ^∗∗^p < 0.01; ^∗∗∗^p < 0.001.

The functional diversity of the LPBN is underscored by a complex anatomical substructure and neurochemical composition (Fulwiler and Saper, 1984). To determine regions of hypoglycemic responsiveness, glucoprivation was pharmacologically induced through administration of the glucose anti-metabolite 2-deoxyglucose (2DG) or insulin (INS) and cFOS-immunoreactivty (IR), a molecular correlate of neuronal activation, assessed across the rostral-caudal extent of the LPBN. Both stimuli increased LPBN cFOS-IR, although the distribution and number of activated neurons was greater upon 2DG administration (Figure 1A–1D; Figure S1 available online). Of particular note was the common induction of cFOS-IR within the superior LPBN (sLPBN) (Figures 1A–1C and 1E). The neuropeptide CCK is highly expressed within the sLPBN (Fulwiler and Saper, 1985), visualized here using a *CCK-ires-Cre::R26-loxSTOPlox-L10-GFP* line (Figures 1F and 1G). 2DG and INS treatment significantly increased cFOS-IR within CCK^LPBN^ neurons (Figures 1H–1K). This identifies CCK^LPBN^ neurons as one neurochemically defined subpopulation of LPBN cells responsive to states of glucoprivation, in addition to other non-CCK-expressing cells. To determine the proximal glucoprivic stimulus to which CCK^LPBN^ neurons are responsive, we assessed their capacity to sense shifts in extracellular glucose concentration. Whole-cell recordings from transgenically labeled CCK^LPBN^ neurons demonstrated that a downward step from 5 mM to 0.5 mM resulted in reversible membrane depolarization in 5/14 cells and in spontaneously firing cells an increase in action potential firing rate (Figures 1L–1N; responding cells defined by a poststimulus response greater than 2×SD ± mean of recorded baseline), establishing these cells as a population of glucose-inhibited neurons.

### CCK^LPBN^ Neuron Activation Induces CR-Like Responses

**Figure 2:**
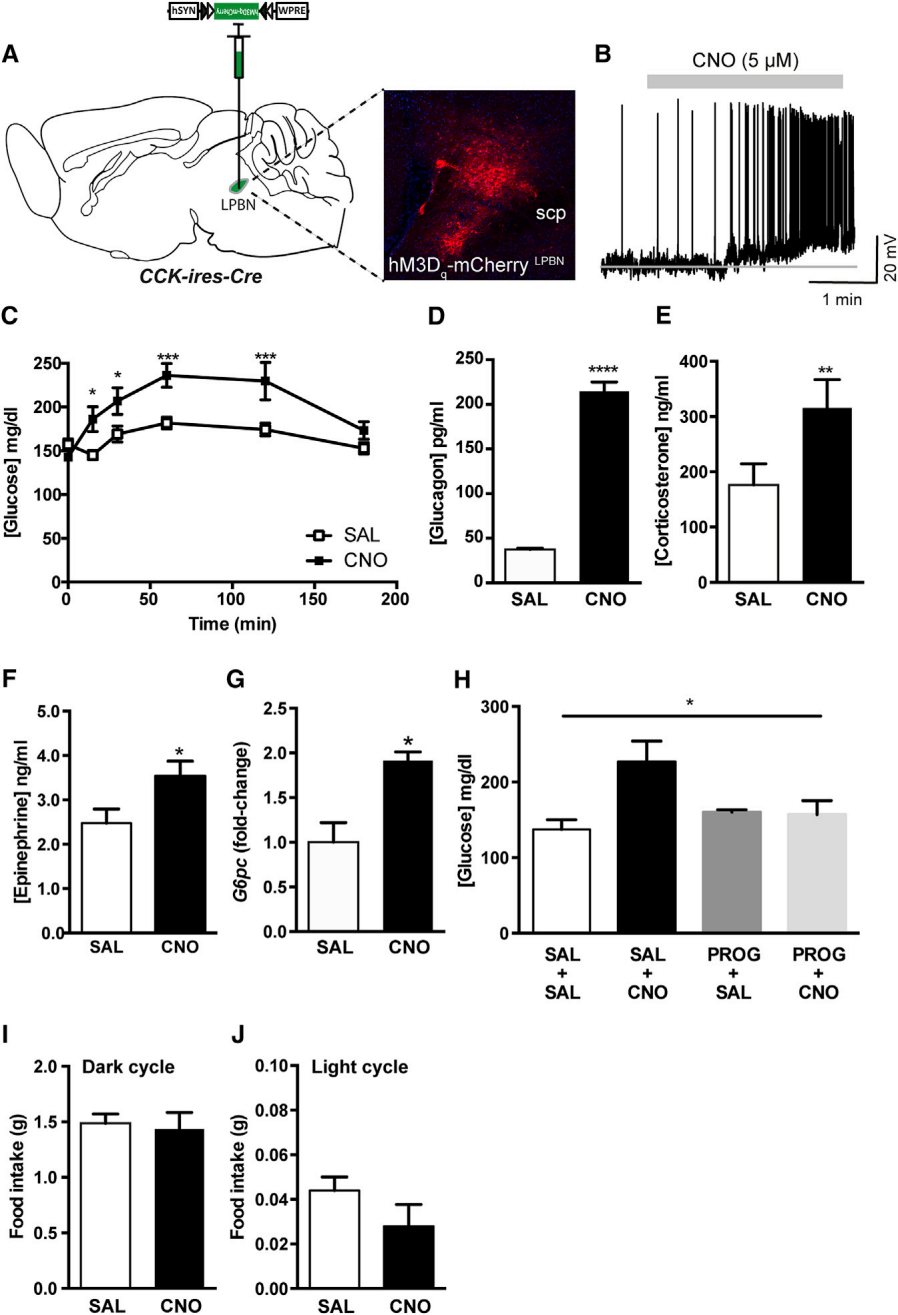
CCK^LPBN^ Neurons Promote SNS-Mediated CR-Like Responses (A and B) Bilateral stereotaxic injection of Cre-dependent excitatory hM3D_q_-mCherry virus into the LPBN of male *CCK-ires-Cre* mice facilitated real-time activation of CCK^LPBN^ neurons. (A) Representative image of Cre-dependent expression of hM3D_q_-mCherry specifically within the LPBN of a *CCK-ires-Cre* mouse. (B) Membrane potential and firing rate of *CCK-ires-Cre*::hM3D_q_-mCherry^LPBN^ neurons increased upon 5 μM CNO application. (C) *CCK-ires-Cre*::hM3D_q_-mCherry^LPBN^ mice exhibited a significant hyperglycemic response to CNO, compared to saline, administration (n = 7; repeated-measures ANOVA, main effect of treatment [F_(1,36)_ = 39.6, p < 0.0001], main effect of time [F_(5,36)_ = 6.6, p = 0.0002], and interaction [F_(5,36)_ = 4.3, p = 0.003]; post hoc comparisons determined by Sidak’s post hoc test for individual time point analysis). (D–F) CNO treatment evoked an increase in serum glucagon (D; n = 3 per group; t test, t_(4)_ = 26.0, p < 0.0001), corticosterone (E; n = 3 to 4 per group; t test, t_(6)_ = 4.4, p = 0.004), and epinephrine concentrations (F; n = 6–8 per group; t test, t_(12)_ = 2.3, p = 0.04). (G) CCK^LPBN^ neuron activation was associated with increased hepatic *G6pc* mRNA expression (n = 3 to 4 per group; t test, t_(4)_ = 2.7, p = 0.05) compared to saline controls. (H) CNO-induced hyperglycemia was abolished by pretreatment with pan-specific CCK-receptor antagonist (20 mg/kg proglumide: PROG) (data shown at 60 min after CNO/SAL administration; n = 5; repeated-measures ANOVA, F_(4,12)_ = 5.0, p = 0.04 with Tukey’s post hoc comparison). (I and J) CNO treatment did not influence feeding behavior compared to saline. (I) Three hour dark-cycle food intake (n = 10; paired t test, t_(9)_ = 0.4, p = 0.7) and (J) 3 hr light-cycle food intake (n = 5; paired t test, t_(4)_ = 1.8, p = 0.1) in ad-libitum-fed mice. CNO, clozapine-*N*-oxide; *G6pc*, glucose-6-phosphatase; scp, superior cerebellar peduncle; SAL, saline. All data are presented as mean ± SEM; ^∗^p < 0.05, ^∗∗^p < 0.01, ^∗∗∗^p < 0.001, ^∗∗∗∗^p < 0.0001.

To probe the relevance of CCK^LPBN^ neurons to glucoregulation, a chemogenetic interrogation of their physiological function using Designer Receptors Activated by Designer Drugs (DREADD) (Alexander et al., 2009) was undertaken. Cre-dependent viral transduction of CCK^LPBN^ neurons with stimulatory hM3D_q_-mCherry facilitated robust activation in response to the designer drug clozapine-*N*-oxide (CNO), as determined by ex vivo slice electrophysiology (Figures 2A and 2B) and in vivo cFOS-IR (Figures S2A and S2B). In freely behaving animals, *CCK-ires-Cre*::hM3D_q_-mCherry^LPBN^ neuron activation prompted an increase in blood glucose concentration that reached a maximal elevation above baseline by 60 min (Figure 2C) (CNO administration had no effect on blood glucose concentration in the absence of DREADD-receptor expression; Figure S2C). Coincident with this rise in blood glucose, CNO administration significantly elevated serum levels of the CR hormones glucagon and corticosterone (Figures 2D and 2E) but had no significant effect on serum INS levels, although there was a trend toward a decrease (Figure S2D). Furthermore, CCK^LPBN^ neuron activation also stimulated sympathoexcitatory drive to the adrenal glands, as indicated by increased serum epinephrine levels (Figure 2F), indicating that these cells couple to the SNS-circuitry requisite for autonomic control of endogenous glucose production. Consistent with these findings, mRNA levels of the glucogenic gene glucose-6-phosphatase (*G6pc*) increased 2-fold in livers of CNO-treated *CCK-ires-Cre::*hM3D_q_-mCherry^LPBN^ mice, compared to saline controls (Figure 2G). We next investigated the necessity of CCK neurotransmission for *CCK-ires-Cre*::hM3D_q_-mCherry^LPBN^-induced hyperglycemia. Systemic pretreatment of *CCK-ires-Cre*::hM3D_q_-mCherry^LPBN^ mice with the CCK-receptor antagonist proglumide abrogated the previously observed elevation in blood glucose (Figure 2H), revealing that LPBN-derived CCK is the functionally relevant neurotransmitter in this physiological context. These data demonstrate that the CCK^LPBN^ neurons, and specifically CCK neuropeptide, are sufficient to drive CR-like responses. Interestingly, despite the involvement of both the LPBN (Wu et al., 2012) and CCK (Little et al., 2005) in appetite control, CCK^LPBN^ neuron activation had no impact on feeding behavior (Figures 2I, 2J, and S2E).

### CCK^LPBN^ Neurons Are Necessary for a Complete CR-Response

**Figure 3:**
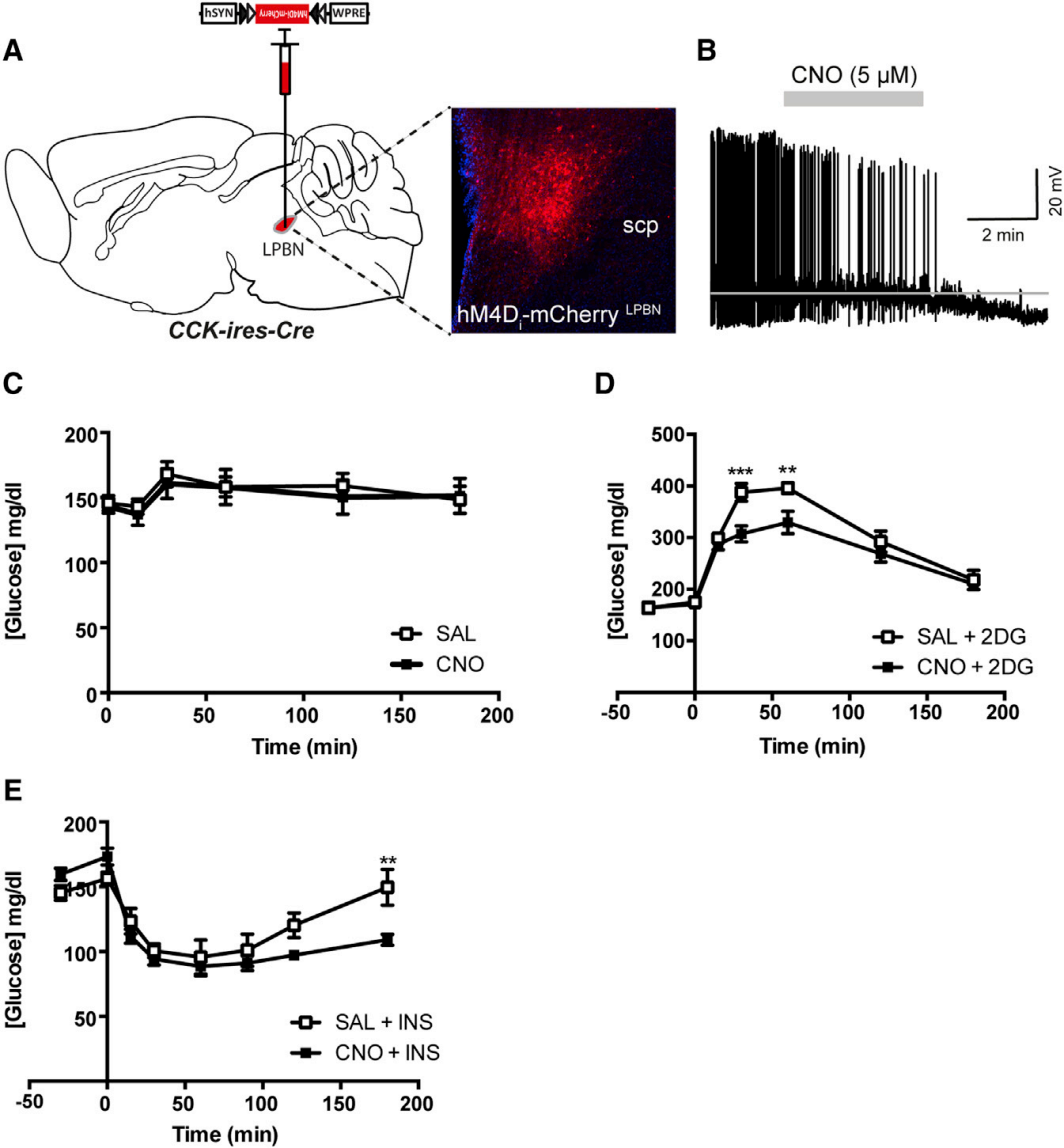
CCK^LPBN^ Neurons Are Necessary for CR-Response to Glucoprivation (A and B) Bilateral stereotaxic injection of Cre-dependent inhibitory hM4D_i_-mCherry virus into the LPBN of male *CCK-ires-Cre* mice facilitated the real-time inhibition of CCK^LPBN^ neurons. (A) Representative image of Cre-dependent expression of hM4D_i_-mCherry specifically within the LPBN of a *CCK-ires-Cre* mouse. (B) Membrane potential and firing rate of *CCK-ires-Cre*::hM4Di-mCherry^LPBN^ neurons decreased upon 5 μM CNO application. (C) CNO-induced CCK^LPBN^ neuron silencing in normoglycemic *CCK-ires-Cre*::hM4D_i_-mCherry^LPBN^ mice had no effect on blood glucose levels, compared to saline controls (n = 11; repeated-measures ANOVA, main effects of treatment and time, and interaction, not significant). (D and E) CNO-induced CCK^LPBN^ neuron silencing under glucoprivic conditions attenuated the CR-response. (D) 2DG-induced CR was significantly diminished by CNO pretreatment compared to saline (n = 8; repeated-measures ANOVA, main effect of treatment [F_(1,49)_ = 14.3, p < 0.0004], main effect of time [F_(6,49)_ = 64.7, p < 0.0001], and interaction [F_(6,49)_ = 2.8, p = 0.02]; post hoc comparisons determined by Sidak’s post hoc test for individual time point analysis). (E) Likewise, INS-induced CR was significantly diminished by CNO pretreatment compared to saline (n = 5; repeated-measures ANOVA, main effect of treatment [F_(1,32)_ = 4.6, p = 0.04], main effect of time [F_(7,32)_ = 22.3, p < 0.0001], and interaction [F_(7,32)_ = 2.8, p = 0.02]; post hoc comparisons determined by Sidak’s post hoc test for individual time point analysis). Abbreviations: 2DG, 2-deoxyglucose; INS, insulin; scp, superior cerebellar peduncle; SAL, saline. All data are presented as mean ± SEM.; ^∗∗^p < 0.01; ^∗∗∗^p < 0.001; ^∗∗∗∗^p < 0.0001.

This ability of CCK^LPBN^ neurons to elevate blood glucose concentration, together with their responsiveness to glucoprivic stimuli, alluded to an involvement in the CR-responses to hypoglycemia, wherein their activation facilitates the re-establishment of normoglycemia. To address the physiological relevance of CCK^LPBN^ neurons to counterregulation, we investigated the consequence of their chemogenetic inhibition within the context of glucoprivation. Ex vivo electrophysiology demonstrated that in the presence of CNO, *CCK-ires-Cre*::hM4D_i_-mCherry^LPBN^ neurons exhibited membrane hyperpolarization and a decrease in action potential firing (Figures 3A and 3B), establishing a capacity to effectively silence CCK^LPBN^ neurons. In freely behaving normoglycemic (ad-libitum-fed) *CCK-ires-Cre*::hM4D_i_-mCherry^LPBN^ mice, CNO administration had no effect on blood glucose concentration (Figure 3C), revealing that these cells are not requisite to the regulation of baseline glycemia. However, CCK^LPBN^ neuron silencing prior to the induction of acute glucoprivation resulted in a significantly diminished CR-response. Specifically, while 2DG administration prompted the expected CR elevation in blood glucose, prior *CCK-ires-Cre*::hM4D_i_-mCherry^LPBN^ neuron inhibition markedly attenuated the hyperglycemic response (Figure 3D). Similarly, under INS-induced hypoglycemia, CCK^LPBN^ neuron silencing engendered an exaggerated hypoglycemic response that impaired re-establishment of normoglycemia (Figure 3E). Thus, CCK^LPBN^ neurons form part of the neurocircuitry underlying centrally regulated glycemia and are both necessary and sufficient for CR-responses to glucoprivation. CCK^LPBN^ neuron silencing did not affect feeding behavior (Figures S3A–S3C).

### CCK^LPBN^ Neuron-Mediated CR Requires Downstream SF1^VMH^ Neurons

**Figure 4:**
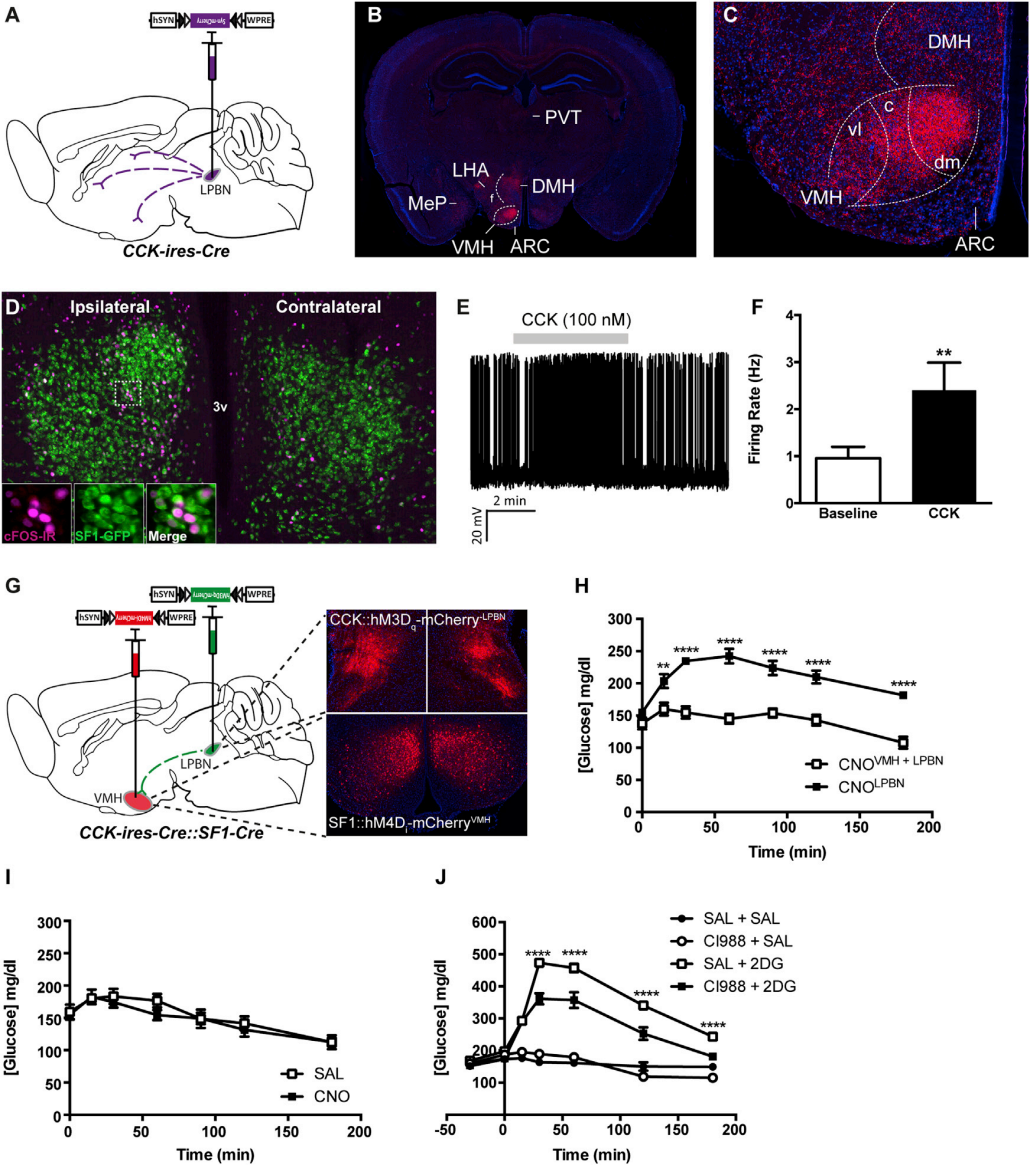
CCK^LPBN^ Neurons Engage SF1^VMH^ Neurons to Mediate CR-Responses (A) Unilateral stereotaxic injection of Cre-dependent synpatophysin-mCherry virus into the LPBN of *CCK-ires-Cre* mice facilitated genetically defined tract tracing of CCK^LPBN^ neuron projections. (B and C) CCK^LPBN^ neurons send ascending projections to the ipsilateral hypothalamus, including the lateral hypothalamus (LH), dorsomedial nucleus (DMH), and VMH. (D) CNO-mediated activation of unilateral *CCK-ires-Cre*::hM3Dq-mCherry^LPBN^ neurons evokes cFOS-IR (magenta) within ipsilateral SF1^VMH^ neurons (green) in *CCK-ires-Cre::SF1-Cre::R26-loxSTOPlox-L10-GFP* mice. (E and F) A total of 55% (5/9) of synaptically isolated SF1^VMH^ neurons were activated by CCK (CCK-8S, 100 nM) in ex vivo slice preparations maintained under hypoglycemic conditions (0.5 mM glucose). (E) Representative electrophysiological trace of a SF1^VMH^ neuron demonstrating CCK-induced activation. (F) CCK-responsive SF1^VMH^ neurons exhibited a 2.5-fold increase in firing frequency over baseline upon CCK-8S administration (n = 6, paired t test, t_(4)_ = 4.1, p = 0.01). (G and H) Functional occlusion of CCK^LPBN^ neuron glucoregulation through concomitant silencing of downstream SF1^VMH^ neurons. (H) *SF1-Cre*::hM4D_i_-mCherry^VMH^ silencing prevents the *CCK-ires-Cre*::hM3D_q_-mCherry^LPBN^-mediated CR-response in CNO-treated double transduced mice, as compared to *CCK-ires-Cre*::hM3D_q_-mCherry^LPBN^ only transduced mice (n = 5 per group; two way ANOVA, main effect of treatment [F_(1,56)_ = 188.2, p < 0.0001], main effect of time [F_(6,56)_ = 11.9, p < 0.0001], and interaction [F_(6,56)_ = 4.5, p = 0.0009]; Sidak’s post hoc test for individual time point analysis). (I) SF1^VMH^ neuron silencing does not influence blood glucose concentrations compared to saline (n = 4; repeated-measures ANOVA; main effects of treatment, time, and interaction not significant). (J) The CR-response to 2DG was significantly attenuated by pretreatment with the selective CCK_B_-receptor antagonist CI988 (n = 5 per group; two-way ANOVA; main effect of treatment [F_(3,112)_ = 369.1, p < 0.0001], main effect of time [F_(6,112)_ = 121.7, p < 0.0001], and interaction [F_(18,112)_ = 36.2, p < 0.001]; post hoc comparisons determined by Tukey’s post hoc test for individual time point analysis). Abbreviations: ARC, arcuate nucleus of the hypothalamus; DMH, dorsomedial nucleus of the hypothalamus; LHA, lateral hypothalamic area; MeP, medial amygdaloid nucleus posterior part; PVT, paraventricular nucleus of the thalamus; SAL, saline; VMH, ventromedial nucleus of the hypothalamus; c, central; vl, ventrolateral; dm, dorsomedial. All data are presented as mean ± SEM; ^∗^p < 0.05; ^∗∗∗^p < 0.001; ^∗∗∗∗^p < 0.0001.

To probe the neurocircuit underlying CCK^LPBN^ neuron-mediated glucoregulation, their specific efferent targets were assessed using genetically encoded neuronal tract-tracing. Unilateral viral transduction of CCK^LPBN^ neurons with Cre-dependent synaptophysin-mCherry revealed a strictly ascending projection profile, with terminals concentrated predominantly within the ipsilateral hypothalamus (Figures 4A–4C and S4A–S4F). The densest site of CCK^LPBN^ neuron innervation was the ventromedial nucleus of the hypothalamus (VMH), and in particular the dorsomedial compartment (dmVMH) (Figure 4C). This robust innervation, together with the well-established glucoregulatory capacity and preautonomic function of the VMH (Borg et al., 1994, Borg et al., 1995, Choi et al., 2013), supported its role as a site of functional outflow for CR CCK^LPBN^ neurons.

SF1 is required for the terminal differentiation of the VMH and is expressed predominantly in the dmVMH of adult mice (Choi et al., 2013). Conditional genetic manipulations have demonstrated that SF1 neurons are required for CR-response to hypoglycemia (Kim et al., 2012, Klöckener et al., 2011, Tong et al., 2007). Thus, we investigated whether SF1^VMH^ neurons form part of a glucoregulatory CCK^LPBN^→VMH microcircuit. Concordant with the lateralized nature of CCK^LPBN^ neuron projections, unilateral *CCK-ires-Cre*::hM3D_q_-mCherry^LPBN^ neuron activation induced an increase in cFOS-IR within transgenically labeled ipsilateral SF1^VMH^ neurons, as compared to the contralateral side, indicating that SF1^VMH^ neurons lie downstream of CCK^LPBN^ neurons (Figure 4D). Consistent with this observation and the excitatory effect of CCK on the VMH (Kow and Pfaff, 1986), electrophysiological recordings from synaptically isolated SF1^VMH^ neurons at low (0.5 mM) and normal (5 mM) glucose conditions revealed that 55% (5/9) were stimulated by exogenous CCK, with responding cells increasing their firing rate 2.5-fold (Figures 4E, 4F, and S4G); importantly, pretreatment of slices with a CCK_B_-receptor antagonist (CI988) blocked responses to CCK in all cells tested (n = 12). Non-SF1^VMH^ neurons did not exhibit a response to CCK (0/12 cells; Figures S4H and S4I). In light of evidence that LPBN-derived CCK is the sole source of CCK terminals within the VMH (Nagai et al., 1987), these data suggest SF1^VMH^ neuron responsiveness to CCK lies in their innervation by CCK^LPBN^ neurons. Furthermore, the robustly glutamatergic nature of the LPBN led us to consider the potential contribution of fast neurotransmission from CCK^LPBN^ neurons. Interestingly, we did not observe VMH projections from glutamatergic vGLUT2^LPBN^ neurons (Figure S4J), and *vGLUT2-ires-Cre*::hM3Dq-mCherry^LPBN^ stimulation failed to elevate blood glucose levels (Figure S4K). These data suggest that VMH-projecting CCK^LPBN^ neurons are not glutamatergic and support our observation that CCK receptor blockade is sufficient to abrogate the hyperglycemic response to CNO in *CCK-ires-Cre*::hM3Dq-mCherry^LPBN^ mice (Figure 2H).

Taking advantage of the spatial exclusivity of CCK and SF1 expression in the LPBN and VMH, respectively, we next generated compound *CCK-ires-Cre::SF1-Cre* mice to facilitate neurochemically explicit manipulation of this CCK^LPBN^→SF1^VMH^ microcircuit. Specifically, we sought to functionally occlude *CCK-ires-Cre*::hM3D_q_-mCherry^LPBN^-induced CR-responses through the simultaneous silencing of downstream *SF1-Cre*::hM4D_i_-mCherry^VMH^ neurons (Figure 4G). Concomitant CNO-induced activation and inhibition of CCK^LPBN^ and SF1^VMH^ neurons, respectively, resulted in abrogation of *CCK-ires-Cre*::hM3D_q_-mCherry^LPBN^-mediated elevation in blood glucose (Figure 4H). Silencing of SF1^VMH^ neurons alone had no effect on blood glucose concentration, indicating a causal interaction between these two populations, and not simply a counteracting additive effect (Figure 4I). Furthermore, like CCK^LPBN^ neurons, silencing of SF1^VMH^ cells within the context of 2DG-glucoprivation induced an attenuated CR-response (Figure S4L). Control experiments performed to rule out any contribution from CCK neurons within the dorsomedial nucleus of the hypothalamus, compact part (cDMH), which neighbors the SF1^VMH^ domain, revealed that CCK^cDMH^ neurons had neither any effect on blood glucose levels nor were engaged by glucoregulatory CCK^LPBN^ neurons (Figures S4M–S4P). These data suggest that the CR function of CCK^LPBN^ neurons is predicated upon their engagement of downstream SF1^VMH^ neurons. Interestingly, systemic antagonism of CCK_B_-receptors, the predominant central receptor isoform (and enriched in the dmVMH), was sufficient to significantly attenuate the hyperglycemic response to 2DG in wild-type mice (Figure 4J), highlighting the salience of CCK_B_-receptor signaling to CR-responses, although not definitively identifying specific receptor-expressing neuronal populations.

Here we demonstrate that CCK^LPBN^ neurons are a population of glucose-sensitive neurons and a requisite component of the neurocircuitry underlying CR-responses to hypoglycemia. Furthermore, the necessity for SF1^VMH^ neurons as downstream effectors of CCK^LPBN^-regulated glycemia befits their established role in CR (Choi et al., 2013, Kim et al., 2012, Tong et al., 2007). In sum, the present study defines the physiological function of a discrete CCK^LPBN^→SF1^VMH^ microcircuit that drives hepatic glucose production and mediates CR responses to hypoglycemia. Furthermore, these data identify a critical function for CCK in the physiological response to glucoprivation. These observations have salience to both homeostatic function in health and dysregulation in disease, in particular diabetes.

## Experimental Procedures

### Animals

*CCK-ires-Cre* (Jackson Laboratories), *SF1-Cre*, *vGLUT2-ires-Cre*, and *R26-loxSTOPlox-L10-GFP* mice were generated and maintained as previously described (Dhillon et al., 2006, Krashes et al., 2014, Taniguchi et al., 2011). Animal care and experimental procedures were performed with approval by the Beth Israel Deaconess Medical Center Institutional Animal Care and Use Committee or were performed in accordance with the UK Animals (Scientific Procedures) Act 1986.

### Viruses

The DREADD viruses used have been described previously: AAV8-hSyn-DIO-hM3D_q_-mCherry and AAV8-hSyn-DIO-hM4D_i_-mCherry (University North Carolina Vector Core) (Krashes et al., 2011). A Cre-dependent expression cassette for hEF1alpha-DIO-synaptophysin-mCherry-WPRE (Opland et al., 2013) was generated (MIT Viral Gene Transfer Core) and the construct packaged in AAV serotype-8 at a titer of 1.3 × 10^13^ vg/ml (Virovek, Inc). Nucleus specific delivery of viruses was achieved through stereotaxic delivery based upon coordinates defined by the Mouse Brain Atlas (Franklin and Paxinos, 2008).

### Blood Glucose Studies

On test days, mice were transferred to new cages and food removed at 9:00 am. Mice remained fasted until 1:00 pm, when the experiment would commence. Blood glucose concentration was determined by tail bleed using a OneTouch Ultra glucometer and test strips (LifeScan, Johnson and Johnson Company). Basal blood glucose concentration was determined prior to injection of any substances and at 15, 30, 60, 120, and 180 min postadministration. Pretreatments were given 30 min prior to pharmacological stimulus. Experiments were within study design (unless otherwise stated), with mice assessed following saline and 1 mg/kg CNO. Animals undergoing loss-of-function experiments involving glucoprivic stimuli (INS or 2DG) were counterbalanced and given 3 weeks recovery time between treatments to ensure normal counterregulatory responses were intact.

### Immunohistochemistry

Brains were sectioned on a freezing microtome at 30 μm. Dual immunofluorescence histochemistry for cFOS-IR, mCherry-IR, and GFP-IR was conducted as previously described (Garfield et al., 2012). Primary antibodies (1/1,000) were as follows: rabbit anti-cFOS (EMD Millipore), rabbit anti-RFP (Clontech Laboratories, Inc.), or chicken anti-GFP (EMD Millipore).

### Serum and Tissue Extraction

For assessment of serum chemistry and hepatic gene expression animals were decapitated, and fresh trunk blood and liver samples were collected. Blood was collected in tubes containing 250 KIU/ml aprotinin (EMD Millipore), allowed to coagulate at room temperature for 30 min, centrifuged at 1,500 × *g* for 10 min, and the resulting serum transferred to a new tube. Samples were flash frozen in liquid nitrogen and kept at −80°C until analyzed. Liver extracts were flash frozen in liquid nitrogen and kept at −80°C until RNA was extracted.

### Electrophysiological Studies

To assess the effects of CNO on CCK^LPBN^ neurons, 5- to 7-week-old *CCK-ires-Cre* mice were injected with either AAV8-DIO-hM3D_q_-mCherry or AAV8-DIO-hM4D_i_-mCherry into the LPBN 3 weeks before recording. Slices were maintained in aCSF containing 10 mM glucose. After acquisition of stable, whole-cell recordings for 2 to 5 min, aCSF solution containing CNO (5 μM) was perfused into the brain slice preparation (for hM4Di-mCherry analysis, cells were injected with 5 pA of current). To assess the effect of CCK on VMH neurons (SF1+ or SF− neurons), 6- to 8-weeks-old *SF1-Cre::R26-loxSTOPlox-L10-GFP* mice were used. CCK-8S (100 nM; Tocris Biosciences) and CI988 (500 nM; Tocris Biosciences) were applied to bath solution containing either 5 mM or 0.5 mM glucose through perfusion. After acquisition of stable, whole-cell recording for 2–5 min, acSF solution containing 100 nM of CCK-8S was perfused for 3 to 5 min. Synaptic blockers (1 mM kynurenate and 100 μM picrotoxin) were added in aCSF to synaptically isolate VMH neurons. The effect of glucose concentration on CCK^LPBN^ neuron activity was assessed using a 5 mM→0.5 mM→5 mM step protocol, with osmolarity adjusted with sucrose.

### Statistics

Statistical analyses were performed using Prism 6 (Graphpad Software, Inc). Data were analyzed using t test, one-way ANOVA, or two-way ANOVA with post hoc comparisons, where appropriate. Data are presented as mean ± SEM, and statistical significance was set at p < 0.05

## Author Contributions

A.S.G. conceived experiments with input from L.K.H., M.G.M., M.L.E., B.B.L., C.M.P., and J.F. A.S.G. performed experiments with help from L.K.H., B.P.S., L.K.B., and J.C.M. Synaptophysin-mCherry construct was made by R.L.N. A.S.G. interpreted data with help from L.K.H., M.L.E., B.B.L., and M.G.M. A.S.G. wrote the manuscript with input from L.K.H., M.L.E., B.B.L., and M.G.M.

## Supplemental Information

Document S1. Figures S1–S4

Document S2. Article plus Supplemental Information
