# Supplementary material for: A Parabrachial-Hypothalamic Cholecystokinin Neurocircuit Controls Counterregulatory Responses to Hypoglycemia
Source: Cell Metab. 2014 Dec 2;20(6):1030–7. doi: 10.1016/j.cmet.2014.11.006 (PMC4261079; doi:10.1016/j.cmet.2014.11.006)

Cell Metabolism, Volume *20*

Supplemental Information

A Parabrachial-Hypothalamic Cholecystokinin Neurocircuit Controls Counterregulatory Responses to Hypoglycemia

Alastair S. Garfield, Bhavik P. Shah, Joseph C. Madara, Luke K. Burke, Christa M. Patterson, Jonathan Flak, Rachael L. Neve, Mark L. Evans, Bradford B. Lowell, Martin G. Myers Jr., and Lora K. Heisler

SUPPLEMENTAL FIGURE LEGENDS

Figure S1, related to Figure 1.

Comparative distribution profile of 2-deoxyglucose and insulin cFOS-IR (red) across the rostral-caudal extent of the LPBN (-5.02 to -5.52 mm from Bregma) compared to saline. Abbreviations: 2DG, 2-deoxyglucose; INS, insulin; SAL, saline; scp, superior cerebellar peduncle; S, superior LPBN; e, external LPBN; c, central LPBN; d, dorsal LPBN; v, ventral LPBN.

Figure S2, related to Figure 2.

(A-B) *CCK-ires-Cre*::hM3D_q-$mCherry^{LPBN}$ neurons (red) exhibited robust cFOS-IR (green) in response to systemic CNO administration (1 mg/kg, i.p.) compared to saline controls. (C) The DREADD-receptor ligand, CNO, has no effect on blood glucose concentration in *CCK-ires-Cre* mice in the absence of Cre-dependent DREADD-receptor expression (n=6, Repeated measures ANOVA, main effect of treatments, time and interaction not significant). (D) Serum insulin levels were not significantly affected by CNO administration in *CCK-ires-Cre*::hM3D_q-$mCherry^{LPBN}$ mice (n=5-8, t-test, $t_{(11)}$=1.4, p=0.2), compared to saline controls (E) *CCK-ires-Cre*::hM3D_q-$mCherry^{LPBN}$ mice do not exhibit a difference in 3 hour food intake following an overnight fast (n=10, t-test, $t_{(9)}$=1.4, p=0.2). All data are presented as mean±SEM.

Figure S3, related to Figure 3.

(A-C) *CCK-ires-Cre*::hM4D_i-$mCherry^{LPBN}$ mice do not exhibit altered feeding behavior in response to CNO administration compared to saline administration. (A) 3 hour dark-cycle food intake in *ad libitum* fed mice (n=7, paired t-test, $t_{(6)}$=0.4, p=0.7). (B) 3 hour light cycle food intake in *ad libitum* fed mice (n=11; paired t-test, $t_{(11)}$=0.4, p=0.7). (C) 3 hour food intake in mice following an overnight fast (n=7; paired t-test, $t_{(6)}$=0.08, p=0.9). All data are presented as mean±SEM.

Figure S4, related to Figure 4.

(A-F) Unilateral stereotaxic injection of Cre-dependent AAV-synpatophysin-mCherry virus into the LPBN of *CCK-ires-Cre* mice facilitated genetically-defined tract tracing of CCK^{LPBN} neuron projections. Only ascending projections were observed, principally terminating within the ipsilateral hypothalamus. (G) CCK-8S application to $SF1^{VMH}$

neurons incubated in normal glucose conditions (5 mM) resulted in an increase in firing frequency (5/9) (paired t-test, $t_{(4)}$=3.24, p< 0.05). (J-K) Non-$SF1^{VMH}$ neurons are unresponsive to CCK in *ex vivo* slice preparations. (H) Non-$SF1^{VMH}$ neurons (10/10) did not exhibit a change in firing frequency over baseline upon CCK administration (paired t-test, $t_{(9)}$=0.1, p=0.9). (I) Representative electrophysiological trace of a CCK insensitive non-$SF1^{VMH}$ neuron. (J) Cre-dependent AAV-synaptophysin-mCherry virus injections into the LPBN of *vGLUT2-ires-Cre* mice revealed no VMH projection. (K) Chemogenetic activation of *vGLUT2-ires-Cre*::$hM3Dq^{LPBN}$ neurons failed to elevate blood glucose levels (n=4, Repeated measures ANOVA, main effect of treatments, time and interaction not significant). (L) Chemogenetic inhibition of *SF1-Cre*::$hM4Di^{VMH}$ neurons resulted in a significant attenuation of the hyperglycemic response to 2DG (n=4; Repeated measures ANOVA, main effect of treatment ($F_{(1,21)}$=43.6, p<0.0004), main effect of time ($F_{(6,21)}$=10.7, p<0.0001) and interaction ($F_{(6,21)}$=3.5, p=0.01), *post-hoc* comparisons determined by Sidak's post-hoc test for individual time point analysis). (M-O) CCK^{cDMH} neurons are not engaged by CCK^{LPBN} neurons. (M) CNO mediated activation of *CCK-ires-Cre*::hM3Dq-$mCherry^{LPBN}$ neurons does not evoke cFOS-IR (magenta) within CCK^{cDMH} neurons (green) in *CCK-ires-Cre::R26-loxSTOPlox-L10-GFP* mice. (N) CCK^{cDMH} neurons are not responsive to exogenous CCK-8S (0/4 cells). (O) chemogenetic silencing of CCK^{cDMH} neurons does not influence blood glucose levels (n=4, Repeated measures ANOVA, main effect of treatments, time and interaction not significant). Abbreviations: ARC, arcuate nucleus of the hypothalamus; AHC, anterior hypothalamic area, central part; BST, bed nucleus of the stria terminalis; DMH, dorsomedial nucleus of the hypothalamus; cDMH, DMH, compact part; LHA, lateral hypothalamic area; LPBN, lateral parabrachial nucleus; MnPO, median preoptic nucleus; MPA, median preoptic area; MeP, medial amygdaloid nucleus; PAG, periaqueductal gray; PVT, paraventricular nucleus of the thalamus; PVH, paraventricular nucleus of the hypothalamus; PP, peripeduncular nucleus; SCh, suprachiasmatic nucleus; SnC, substania nigra pars compacta; VMH, ventromedial nucleus of the hypothalamus. All data are presented as mean±SEM; *p<0.05, **p<0.01, ***p<0.001.

SUPPLEMENTAL EXPERIMENTAL PROCEDURES

Animals

Mice were housed at 22°C–24°C with a 12-hour light/12-hour dark cycle and standard mouse chow (Teklad F6 Rodent Diet 8664) and water provided *ad libitum*. Animal care and experimental procedures were performed with approval by the Beth Israel Deaconess Medical Center Institutional Animal Care and Use Committee or were performed in accordance with the UK Animals (Scientific Procedures) Act 1986.

Drugs

Drugs for *in vivo* use were prepared in sterile saline and administered intraperitoneally (i.p.). Clozapine-*N*-oxide (CNO; a generous gift from Dr Bryan Roth, University of North Carolina) was administered at 1 mg/kg, humalin R (Eli Lilly) was administered at 1 U/kg, 2-Deoxyglucose (2DG, Sigma Aldrich) was administered at 500 mg/kg, proglumide (Tocris Bioscience) was administered at 20 mg/kg. CI988 (Tocris Bioscience) was dissolved in 50% DMSO and administered at 1 mg/kg.

Stereotaxic surgery

Nucleus specific delivery of AAVs was achieved through stereotaxic injection, as previously described (Krashes et al., 2011). In brief, 5-8 week old male mice were anesthetized with 100 mg/kg ketamine and 10 mg/kg xylazine (i.p). Mice were placed in a stereotaxic frame (Kopf Instruments) and the skull exposed by a single longitudinal incision. A hole was drilled in the skull at the injection site and a virus filled pulled-glass micropipette (diameter 20-40 µm) inserted into the brain. Virus was delivered under air pressure using a micromanipulator (Grass Technologies). For LPBN injections 25 nl and for VMH 50 nl of virus was delivered. The pipette remained in place for a minimum of 5 minutes after injection. Based on the Mouse Brain Atlas (Franklin and Paxinos, 2008) the following coordinates used for targeting (mm from Bregma): LPBN, A/P, -5.02; M/V, ±1.3; D/V, -3.4 mm. VMH, A/P, -1.57; M/V, ± 0.3; D/V, -5.60 mm. Animals were administered an analgesic (5 mg/kg Meloxicam, Norbrook) for 3 days post-operatively and given a minimum of 14 days recovery before being used in any experiments, during this time they were also acclimated to i.p injection by once daily injections of 0.3 ml sterile saline. Mice injected with AAV8-hSyn-DIO-Synaptophysin-mCherry were tested at least 3 weeks after treatment to ensure labelling of distal projection sites.

Brain tissue preparation

Mice were terminally anesthetized with chloral hydrate (Sigma Aldrich) and transcardially perfused with phosphate-buffered saline (PBS) followed by 10% neutral buffered formalin (Fisher Scientific). Brains were extracted, cryoprotected in 20% sucrose, and sectioned coronally on a freezing sliding microtome (Leica Biosystems) at 30 µm and collected in four equal series.

Immunohistochemistry

Briefly, sections were washed in PBS before blocking in 0.5% BSA/0.25% Triton X-100 in PBS for 1 hour at room temperature. Tissue was incubated overnight at room temperature in blocking buffer containing the primary antibodies (diluted 1/1000): rabbit anti-cFOS (EMD Millipore), rabbit anti-RFP (Clontech Laboratories, Inc.) or chicken anti-GFP (EMD Millipore). The next day sections were washed in PBS then incubated in blocking buffer containing appropriate secondary antibody (1/1000, Alexa Fluor; Life Technologies) for 1 hour. Sections were mounted onto microscope slides and coverslipped in an aqueous mounting medium containing DAPI (Vectastain; Vector Laboratories). Slides were imaged on a VS120 slide scanner (Olympus). For counting of cFOS-IR nuclei, the boundaries of the nucleus were defined using neuroanatomical landmarks and the Mouse Brain Atlas (Franklin and Paxinos, 2008). All sections within one series containing the nucleus of interest were counted bilaterally and an average determined from each section. For CCK co-localisation the total number of CCK^{LPBN} neurons and number of cFOS-IR+CCK^{LPBN} neurons in one series were counted.

Blood chemistry analysis

Insulin and glucagon levels were determined from serum samples recovered 60 minutes after SAL or CNO administration by means of a multiplex ELISA (EMD Millipore). Catecholamine levels were determined from plasma samples recovered 60 minutes after SAL or CNO administration by HPLC via electrochemical detection. Specifically, trunk blood was collected in tubes containing 236 mM EGTA (Sigma Aldrich) and 195 mM glutathione (Sigma Aldrich) following decapitated. Samples were centrifuged for 15 minutes at 1,500g at 4°C and the resulting plasma transferred to a new tube. Plasma was absorbed onto alumina at a pH 8.6, eluted with dilute perchloric acid and auto-injected onto a C18 reversed-phase column. An internal standard (dehydroxylbenzylamine; DHBA) was included with each extraction to monitor recovery

and standard curves for both epinephrine and norepinephrine were run. Results are quantitated through a chromatography data station.

RNA extraction and quantitative-PCR

Total RNA was extracted from 100 mg of liver using PureLink RNA mini kit (Life Technologies), as per the manufacture's instructions. Multiplex qPCR analysis of glucose-6-phosphatase (*G6pc*) and 18S ribosomal RNA (18S) expression was conducted using pre-designed Taqman assays and Taqman One-Step RT-PCR Master Mix (Applied Biosystems), as per the manufacture's instructions. qPCR was run on a Applied Biosystems Prism 7000 Sequence Detection System. Reactions were multiplexed for G6pc/18S. Data were analyzed using the 2-ΔΔCT method.

Feeding studies

Dark-cycle food intake. Mice were injected with saline or 1 mg/kg CNO 30 minutes prior to the onset of the dark cycle and food removed. Food was returned at lights-off and weighed every hour over the next three hours. *Post-fast reefed.* Mice were fasted overnight. The following morning, mice were injected with saline or 1 mg/kg CNO and food returned 30 minutes later and weighed every hour for the next three hours. *Light-cycle food intake*. Mice were injected with saline or 1 mg/kg CNO at 9am and food intake monitored over the next three hours.

Electrophysiology

For brain slice preparation, 5-12 week old mice were terminally anesthetized with 7% chloral hydrate (i.p) and transcardially perfused with ice-cold artificial cerebral spinal fluid (aCSF). The mice were decapitated and brains were removed. Brains were immediately submerged in ice-cold, carbogen-saturated (95% O_2, 5% CO_2) high sucrose solution (238 mM sucrose, 26 mM $NaHCO_3$, 2.5 mM KCl, 1.0 mM NaH_2PO_4, 5.0 mM $MgCl_2$, 10.0 mM $CaCl_2$, 10 mM glucose). 300 μM thick coronal sections were cut with a Leica VT1000S vibratome and incubated in oxygenated aCSF (126 mM NaCl, 21.4 mM $NaHCO_3$, 2.5 mM KCl, 1.2 mM NaH_2PO_4, 1.2 mM $MgCl_2$, 2.4 mM $CaCl_2$, 10 mM glucose) at 34˚C for 30 minutes. Slices were maintained and recorded at room temperature (20–24˚C). The intracellular solution for current clamp recordings contained: 128 mM K gluconate, 10 mM KCl, 10 mM HEPES, 1 mM EGTA, 1 mM $MgCl_2$, 0.3 mM $CaCl_2$, 5 mM Na_2ATP and 0.3 mM NaGTP, adjusted to pH 7.3 with KOH. All recordings were made

using multiclamp 700B amplifier, and the data was filtered at 2 kHz and digitized at 10 kHz. For low glucose conditions, cells were incubated in oxygenated aCSF containing 0.5 mM glucose, with osmolarity adjusted with sucrose. In all studies responding cells were defined by a stimulus induced change in firing rate or membrane potential that was 2xSD±mean.

Supplemental Figure 1

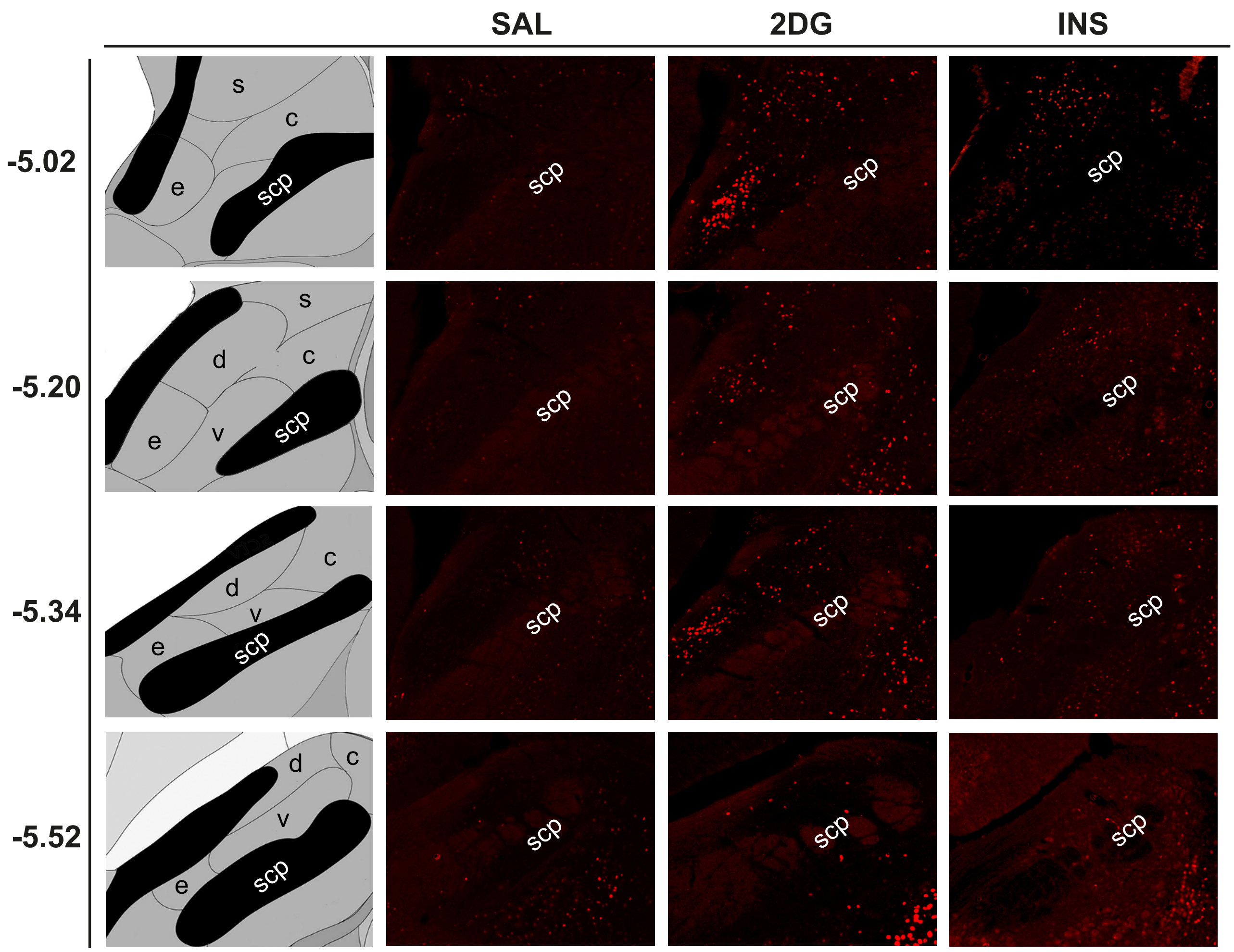

Supplemental Figure 2

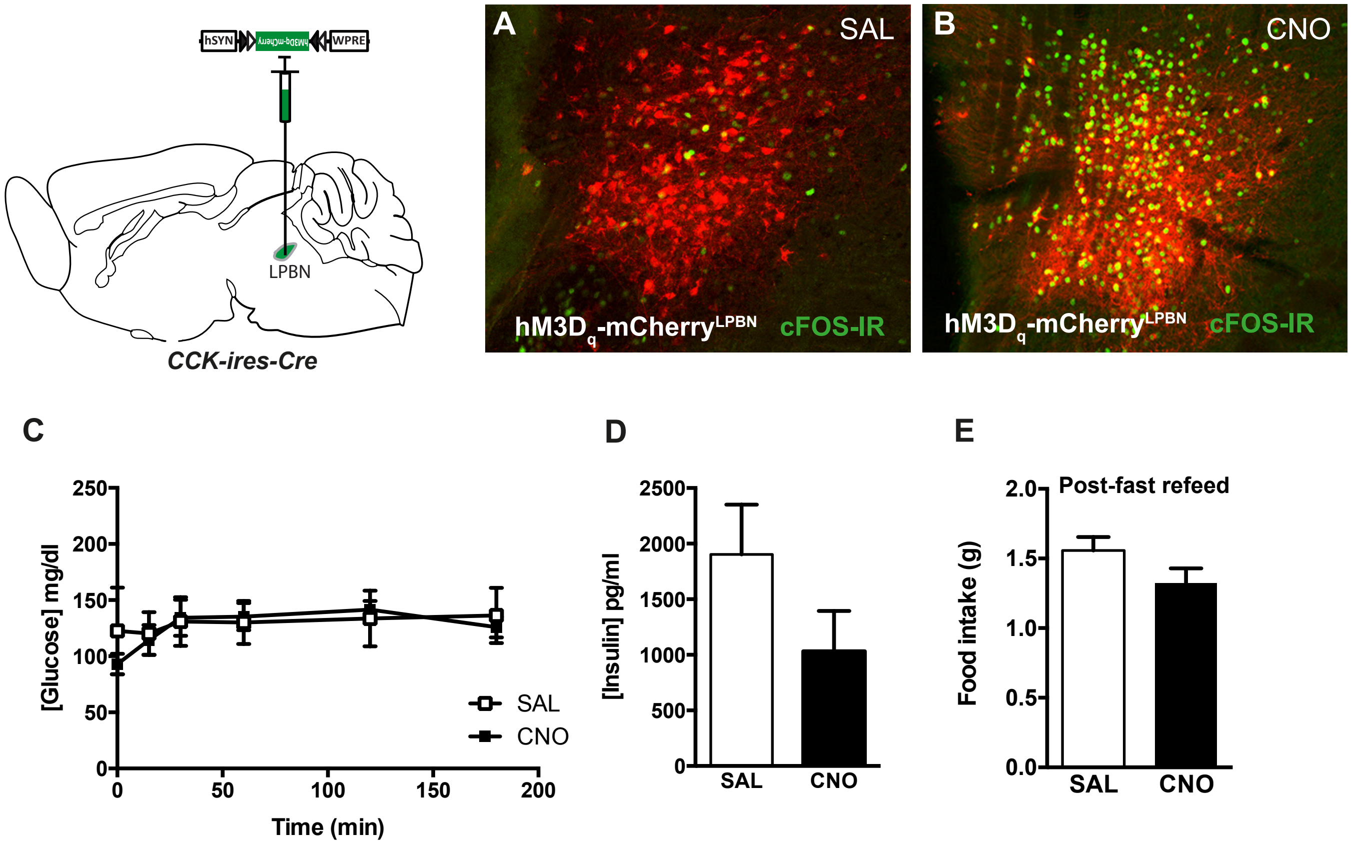

Supplemental Figure 3

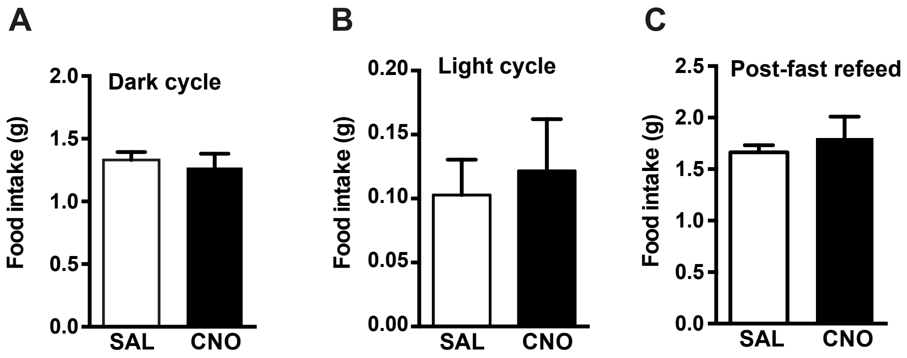

Supplemental Figure 4

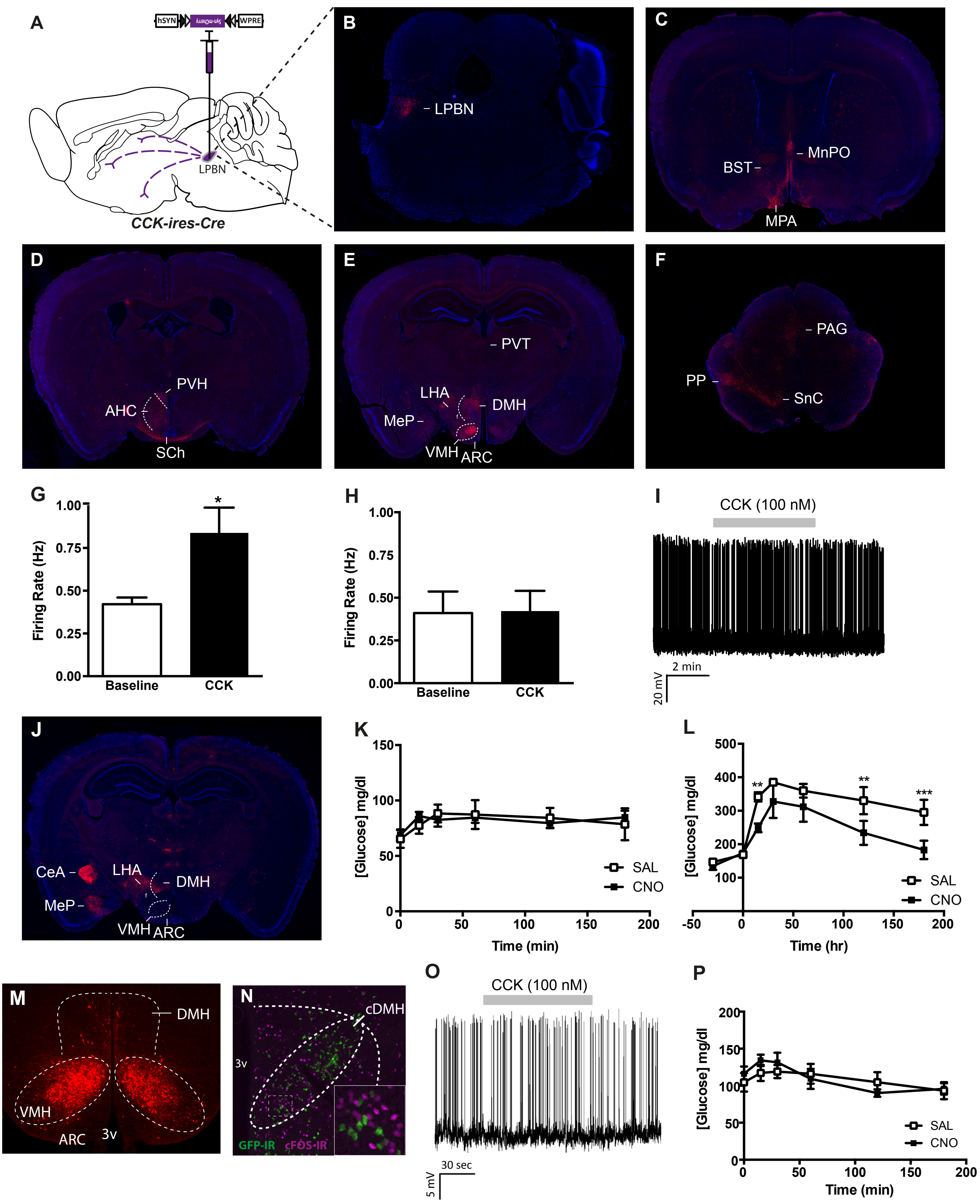

Supplement: Document S1. Figures S1–S4 [file mmc1.pdf]
